# Detection of *Leishmania* spp. in Small Non-Flying Mammals (Didelphimorphia and Rodentia) from Bahia, Northeast Brazil

**DOI:** 10.3390/ani15040588

**Published:** 2025-02-18

**Authors:** Graziela Baroni de Souza, Hllytchaikra Ferraz Fehlberg, Beatris Felipe Rosa, Cássia Matos Ribeiro, Anaiá da Paixão Sevá, Bianca Mendes Maciel, Martin Roberto Del Valle Alvarez, George Rêgo Albuquerque, Fabiana Lessa Silva

**Affiliations:** 1 Postgraduate Programme in Animal Science, Hospital Veterinário (Veterinary Hospital), Universidade Estadual de Santa Cruz (State University of Santa Cruz)—UESC, Soane Nazaré de Andrade Campus, Ilhéus 45662-900, BA, Brazil; grazielabaroni@hotmail.com (G.B.d.S.); ferrazhellen@hotmail.com (H.F.F.); 2 Postgraduate Programme in Zoology, Universidade Estadual de Santa Cruz (State University of Santa Cruz)—UESC, Soane Nazaré de Andrade Campus, Ilhéus 45662-900, BA, Brazil; beatris.rosa@hotmail.com; 3 Graduation in Veterinary Medicine, Universidade Estadual de Santa Cruz (State University of Santa Cruz)—UESC, Soane Nazaré de Andrade Campus, Ilhéus 45662-900, BA, Brazil; cassia.matos@hotmail.com; 4 Department of Agrarian and Environmental Sciences, Universidade Estadual de Santa Cruz (State University of Santa Cruz)—UESC, Soane Nazaré de Andrade Campus, Ilhéus 45662-900, BA, Brazil; 5 Department of Biological Sciences, Universidade Estadual de Santa Cruz (State University of Santa Cruz)—UESC, Soane Nazaré de Andrade Campus, Ilhéus 45662-900, BA, Brazil

**Keywords:** leishmaniasis, wild mammals, reservoir, real-time polymerase chain reaction

## Abstract

This study aimed to identify *Leishmania* species in small non-flying mammals captured in semi-deciduous forest fragments of the Atlantic Forest and pastures in the Southwest region of Bahia state, Northeast Brazil. A total of 445 animals belonging to 11 different species were captured, the majority being rodents (75.7%; 337), followed by marsupials (24.2%; 108), and the most prevalent species were *Cerradomys vivoi*, *Calomys expulsus*, *Necromys Lasiurus,* and *Marmosops incanus.* Liver, spleen, kidney, heart, and lung fragments were collected for subsequent molecular diagnosis. *Leishmania* spp. kDNA amplification in positive samples was performed using real-time polymerase chain reaction (qPCR). Species identification of *Leishmania* was conducted through nested PCR, followed by sequencing. *Leishmania* spp. infection was detected in 2.92% (13/445) of the animals. Sequencing revealed that *L. infantum* infected three animals, while the species of the agent in the other animals could not be determined. The results indicate the presence of *Leishmania* spp. in the studied region, primarily affecting the local wildlife. These findings not only highlight the risk of transmission to domestic animals and humans in close contact with forest remnants, but also underscore the critical role of these fragments in supporting native fauna. However, it is worth noting that the continuous deforestation of these forest remnants could lead to increased contact between wildlife, domestic animals, and humans, thereby elevating the risk of transmission.

## Simple Summary

This study aimed to identify *Leishmania* species in small non-flying mammals captured in semi-deciduous forest fragments of the Atlantic Forest and pastures in the Southwest region of Bahia state, Northeast Brazil. A total of 445 animals belonging to 11 different species were captured. Liver, spleen, kidney, heart, and lung fragments were collected for subsequent molecular diagnosis. *Leishmania* spp. kDNA amplification in positive samples was performed using real-time polymerase chain reaction (qPCR). Species identification of *Leishmania* was conducted through nested PCR, followed by sequencing. *Leishmania* spp. infection was detected in 2.92% (13/445) of the animals. Sequencing revealed that *L. infantum* infected three animals, while the species of the agent in the other animals could not be determined. The results indicate the presence of *Leishmania* spp. in the studied region, primarily affecting the local wildlife. These findings not only highlight the risk of transmission to domestic animals and humans in close contact with the remaining forest, but also underscore the critical role of these fragments in supporting native fauna. However, it is worth noting that the continuous deforestation of these forest remnants could lead to increased contact between wildlife, domestic animals, and humans, thereby elevating the risk of transmission.

## 1. Introduction

Parasitic zoonoses are ubiquitous diseases in several vertebrate species and cause a major impact on public health, generating economic losses in several countries around the world [1,2]. Many of these diseases are caused by intestinal parasites such as those from the Taeniidae family, for example, and others are vector-borne, such as *Trypanosoma cruzi* and *Leishmania* spp.

Leishmaniases are anthropozoonoses of worldwide importance that are included in the group of neglected tropical diseases and have an important impact on public health since they affect various domestic and wild mammals, including humans [3]. These diseases are caused by more than 20 intracellular parasite species belonging to the genus *Leishmania* [4]. More than 30 species of female phlebotomines are considered vectors and transmit the agent during blood meal in the host [4,5]. 

The occurrence of the disease in humans and domestic animals is the consequence of ecological disturbances and alterations in the epidemiological cycle of the parasite [6]. An example of this is alteration to the habitat of the vector, which was primarily found in wild environments; however, due to the intense anthropization of the forests, it has been found in intradomiciliary and peridomiciliary environments [6,7,8,9].

According to the World Health Organization (WHO) [10], it is currently estimated that over a billion people are at risk of being infected by *Leishmania* spp. The disease clinically manifests in two forms, visceral leishmaniasis (VL) and cutaneous leishmaniasis (CL), depending on the parasite species involved in the infection and the defenses of the host [3]. In the Americas, the species *L. infantum* causes VL [11], while in Brazil, the species causing CL are *Leishmania (Leishmania) amazonensis, Leishmania (Viannia) braziliensis*, *Leishmania (Viannia) guyanensis, Leishmania (Viannia) shawi, Leishmania (Viannia) lainsoni, Leishmania (Viannia) lindenbergi*, *Leishmania (Viannia) naiff, and Leishmania (Viannia) panamensis* [3].

The variety of *Leishmania* hosts, vectors, and species, together with intense deforestation, forest fragmentation, urbanization of new areas, and climate change, hinder the control and prevention of leishmaniases [3,12]. Although knowledge of this disease has improved in recent decades, little is known regarding the spectrum of host mammal species and parasite vectors in nature [12,13].

Small rodents and marsupials are distributed across different biomes, with a richness and abundance of these species [14,15,16]. This wide variety of species and their respective exemplars foster the greater interaction of these animals with *Leishmania* spp. in their habitats, which makes them important sentinel species contributing to the impacts that occur in the wild [17].

The assessment of the occurrence of *Leishmania* spp. infection in small wild mammals provides critical insight into parasite–host interactions and defines the role of these animals in the transmission cycle of the agent [17]. In Brazil, the state of Bahia is considered endemic for CL and VL [18,19]. In the period from 2007 to 2022, the Brazilian Northeast was the region with the highest incidence of VL (28,544 cases) and the second highest incidence of CL (91,609 cases) in the country, which is equivalent to 53.39% and 28.84% of the total confirmed cases, respectively [20]. Among the nine northeastern states, Bahia is in the third (4574 cases) and first (42,130 cases) position in relation to the number of confirmed and reported cases of VL and CT, respectively, which demonstrates the expansive endemic nature of the disease in this location [18,19,20]. However, studies on this disease involving small mammals in the region remain scarce. The Southwest region of Bahia has an area of Atlantic Forest in which various species of such individuals can be found in abundance. However, no studies have been found on wild rodents and marsupials infected by the parasites in this region. Thus, this study aimed to investigate the occurrence of *Leishmania* spp. infection and characterize the species involved in small rodents and marsupials collected from the remnants of the Atlantic Forest located in the municipalities of Itapetinga and Itambé, in Southwest Bahia.

## 2. Material and Methods

### 2.1. Study Area

This study is part of a broader project titled “Small non-flying mammals in south Bahia: learning about the mastofauna of Bahia” (PROPP 00220.1100.1645, ICMBio License 17131-4), which predetermined the study area, animal capture methods, and biological sample collection.

The areas selected for the study are located in Itapetinga and Itambé, in the Southwest region of the state of Bahia. The main economic activity of these municipalities is livestock farming, based on the extensive system [21]. The biome is represented by the Atlantic Forest and the climate is tropical, with a mean annual temperature of 23.2 °C, mean annual rainfall of 816 mm, and dry winter and rainy summer, according to the Köppen–Geiger classification [22].

**Figure 1:**
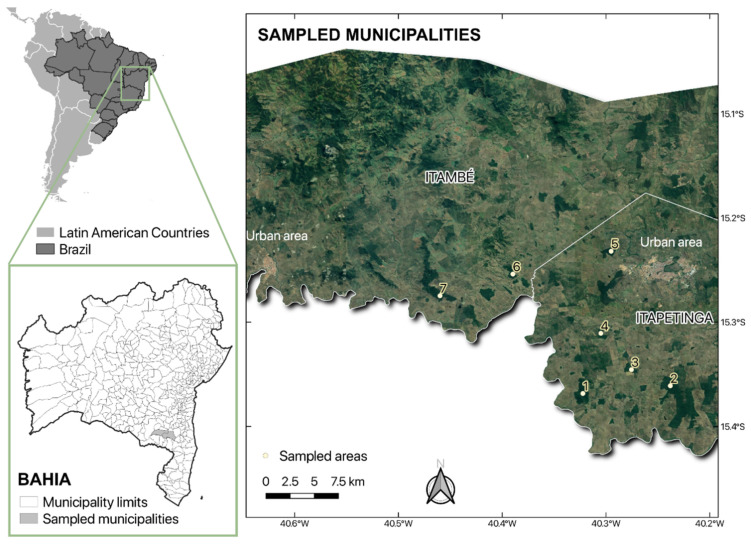
Sampling areas of rodents and marsupials collected from forest remnants in Southwest Bahia, Brazil. Graphic data: Instituto Brasileiro de Geografia e Estatística (Brazilian Institute of Geography and Statistics) (IBGE) and the Instituto Nacional de Pesquisas Espaciais (National Institue for Space Research) (INPE). Preparation: Sevá, A. P. (2022).

The sampled areas were georeferenced using the Global Positioning System (GPS) and their geographic coordinates are shown in Figure 1.

### 2.2. Animal Capture

The animals were collected according to ethical guidelines [23]. The research methodology was approved by the Animal Ethics and Well-Being Committee of the Santa Cruz State University, under protocol CEUA 028/18.

The animals were captured in two seven-day campaigns, the first in August 2018 (dry season) and the second in February 2019 (rainy season). The traps used were Sherman^®^ (31 × 8 × 9 cm) and Tomahawk^®^ (50 × 21.5 × 20 cm) live traps (75 traps/area) and pitfall traps (4 buckets/area).

The live traps were inspected daily in the morning and baited with a mixture of peanut candy, sardines in soybean oil, ripe bananas, cornmeal, and oat flakes. The bait was replenished as needed. The total sampling effort for both dry and rainy seasons amounted to 7350 live traps per night.

The pitfall traps were installed in the forest fragment in each sampling area/unit, each containing four 60 L buckets arranged in a line and buried 10 m apart in the soil, connected by plastic canvasses. The bottoms of the buckets were perorated to prevent water buildup on rainy days. The pitfall traps remained open for seven consecutive nights and were inspected every morning, totaling a sampling effort at the end of the two campaigns of 392 buckets per night. All the traps were uninstalled at the end of each campaign and duly washed.

### 2.3. Biological Material Collection

The captured animals were transported in the traps to the field laboratory facility for sample handling and collection. Ear tags were used to identify and mark juveniles and pregnant and lactating females before releasing them back to their original capture sites.

Adult males and non-gestating females were euthanized using ketamine hydrochloride (30 mg/kg for marsupials and 100 mg/kg for rodents) with xylazine hydrochloride (2 mg/kg for marsupials and 5 mg/kg for rodents), as indicated by Sikes et al. [23]. Subsequently, samples of liver, spleen, kidney, heart, and lung were collected, individually stored in cryogenic tubes, identified, and preserved at a temperature of −20 °C for subsequent molecular analysis. Other tissues were not collected for this study. The specimens were the object of study of the research project mentioned above (PROPP 00220.1100.1645, ICMBio License 17131-4), being deposited as control material in the Alexandre Rodrigues Ferreira mammal collection belonging to the State University of Santa Cruz (CMARF/UESC).

### 2.4. DNA Extraction

For each animal, DNA was extracted based on a pool of samples (liver, spleen, kidney, heart, and lung), using an Easy-DNA kit (Invitrogen^®^, Carlsbad, 5781 Van Allen Way, CA, USA), according to the manufacturer’s instructions. Subsequently, the DNA concentration of each sample was quantified using NanoDrop 2000 (ThermoScientific, 68 3rd Ave, MA, USA) and the DNA was then stored at a temperature of −20 °C until molecular analysis.

DNA was also extracted from the cultures of *L. amazonensis* (strain IFLA/BR 1967/PH8). The promastigote cells present in 1 mL of culture were counted prior to extraction using a Neubauer chamber under a common optical microscope, following conventional methodology. Subsequently, the DNA was extracted as previously described.

### 2.5. DNA Detection of Leishmania spp. Using Real-Time PCR (qPCR)

For DNA amplification of *Leishmania* spp., primers 13A (5′-GTG GGG GAG GGG CGT TCT-3′) and 13B (5′-ATT TTA CAC CAA CCC CCA GTT-3′) were used, as they amplify a fragment with 120 pairs of bases of a kDNA region of the genus *Leishmania* [24]. Amplification was carried out on an AB 7500 Fast thermocycler (Applied Biosystems) using the SYBR^®^ Green (Promega, 2800 Woods Hollow Rd, Madison, WI, USA) system. The DNA extracted from the *L. amazonensis* culture was used as positive control.

The reaction was standardized at a final volume of 20 µL, containing 10 µL of GoTaq^®^ qPCR Master Mix (Promega); 1 µL of each primer at 5 µM; 0.1 µL of CXR Reference Dye (Promega); and 4 µL of DNA at 50 ng/µL. The amplification conditions were a cycle of 50 °C for 2 min, a cycle of 95 °C for 10 min, 45 cycles of 95 °C for 15 s, and 60 °C for 1 min. The dissociation curve consisted of a cycle of 95 °C for 15 s (ramp of 1.6 °C/s), 60 °C for 1 min (ramp of 1.6 °C/s), and 95 °C for 15 s (ramp of 0.15 °C/s).

### 2.6. Identification of Leishmania Species by Nested PCR

The positive samples in the real-time PCR were subjected to nested PCR based on SSU rDNA. The amplifications were carried out on a ProFlex PCR System thermocycler (Applied Biosystems by Life Technologies, 35 Wiggins Ave, Bedford, MA, USA) and the final volume of the reactions was standardized to 25 µL, containing 2.5 µL of buffer; 2 mM of MgCl_2_; 0.2 mM of dNTPs; 0.2 µM of each primer at 5 µM; 2U of Platinum^TM^ Taq DNA Polymerase (Invitrogen^TM^); and 5 µL of genome DNA [25]. The first PCR was carried out with primers S4 (GAT CCA GCT GCA GGT TCA CC) and S12 (GGT TGA TTC CGT CAA CGG AC), as described by Uliana et al. [26]. The thermocycle conditions were 94 °C for 3 min, followed by 35 cycles at 94 °C for 1 min, 50 °C for 1 min, and 72 °C for 1 min, and a final extension at 72 °C for 7 min.

The 520 pb fragment obtained from the first PCR reaction was used as DNA in the nested PCR, using primers S17 (CCAAG CTGCC CAGTA GAAT) and S18 (TCGGG CGGAT AAAAC CC), specific for the genus *Leishmania*, which amplify a fragment of 490 pb. The final volume of the reaction and the reagents used were the same as those previously described. The thermocycle conditions were 94 °C for 4 min, followed by 30 cycles at 94 °C for 1 min, 55 °C for 1 min, and 72 °C for 30 s, and a final extension at 72 °C for 7 min [25].

DNA extracted from the *L. amazonensis* culture (IFLA/BR 1967/PH8) was used as positive control in the reactions and ultra-pure water was used as negative control. The PCR products were subjected to electrophoresis in agarose gel at 2.0% containing Sybr Green (Invitrogen^®^). Band presence was analyzed using a transilluminator (Loccus Biotecnologia, 836 Santa Monica, Cotia, São Paulo, Brazil).

### 2.7. Species Dentification of Leishmania Species Using DNA Sequence Analyses

The amplification products of each sample were purified with a PureLink^®^ Genomic DNA kit (Invitrogen) and sent to Fiocruz (Oswaldo Cruz Foundation) (Salvador, Bahia, Brazil) for sequencing and subsequent comparison with the rDNA sequence of reference strains of *Leishmania* (L.) *amazonensis*, *Leishmania (Viannia)* sp., and *Leishmania* (L.) *infantum chagasi*, as described by Uliana et al. [27]. Sequencing was carried out on the ABI-PRISM 3100 Genetic Analyzer platform (Applied Biosystems) in both directions. The primers used were S17 and S18, which are specific for the genus *Leishmania*, and that amplify a fragment of 490 pb [25]. The chromatograms were analyzed using FinchTV 1.4.0 software. The sequences obtained were deposited in GenBank (accession numbers PQ585730, PQ585731, and PQ585732).

### 2.8. Statistical Analysis

The species, individuals, and orders of animals infected and non-infected with *Leishmania* spp. were compared using the Chi-squared test. The comparison of prevalences between areas with positive animals was also performed by using Chi-squared test. These tests were chosen according to the data distribution. Confidence intervals of 95% of the prevalences of *Leishmania* spp. infection by species, orders, and areas were calculated. For comparison between the distances of the areas with and without positive animals for *Leishmania* spp. to anthropized areas (urban centers), the Wilcoxon test was carried out after definition of non-normal distribution using the Shapiro–Wilk normality test. For all the analyses, a significance level of *p* < 0.05 was considered and they were performed on the R program, version 4.2.0.

## 3. Results

A total of 445 animals were collected, belonging to 11 different species, the majority being rodents (75.7%; 337), followed by marsupials (24.2%; 108), as demonstrated in Table 1. Most of the animals in this study were collected during the dry season (88.7%; 395/445) and the most prevalent species in the regions of Itambé and Itapetinga were *Cerradomys vivoi*, *Calomys expulsus*, *Necromys Lasiurus,* and *Marmosops incanus* (Table 1).

Of the 445 animals collected, 13 were found to be naturally infected with *Leishmania* spp. according to the qPCR, representing a prevalence of 2.9% (13/445; CI 95%: 1.7–4.9%). The animals that were positive for *Leishmania* spp. were found in areas 1, 6, and 7, which are the furthest away from the urban centers (Table 2). The mean prevalence of infection in the areas where there were positive animals was 6.58%, ranging from 4.34% to 10%, and there was no statistical difference in the prevalence between them (X^2^ = 2.02, *p* value = 0.36). With the exception of area 2, it was found that all the studied areas are anthropized, with constant deforestation for timber, in addition to being surrounded by farms, with houses and domestic animals (dogs, horses, and cattle) often freely raised with access to the forest.

However, the areas with positive animals were furthest away from the urban centers (median of 11 km), with a significant difference (W = 12, *p* = 0.04975) in relation to the closest areas (median = 7.25 km), with only negative animals (Table 2).

The 13 positive animals belonged to the species *N. lasiurus* (1/74) and *G. microtarsus* (12/24), representing a prevalence of 13.26% (13/98; CI: 7.9–22.0), with a significant difference between the two species (X^2^ = 33.17, *p* < 0.001) (Table 3). The sequencing enabled the detection of three marsupials from the species *G. microtarsus* infected with *L. infantum* (Table 4).

## 4. Discussion

Leishmaniases are among the parasitic diseases with the greatest impact on human beings, being considered a neglected tropical disease (NTD); however, there is still no clear understanding regarding the mammalian hosts of most *Leishmania* species, which highlights the importance of studies that identify these parasites in mammalian species other than those commonly reported as reservoirs [13,28,29].

The rodent *N. lasiurus* is widely distributed in Brazil [14] and various studies have reported infections of this species with *L. braziliensis*, *L. amazonensis*, and *Leishmania* spp. in the states of Pernambuco, Mato Grosso, Minas Gerais, and the Distrito Federal (Federal District) [17,30,31,32,33].However, the present study is the first to report *Leishmania* spp. infection in this rodent in the state of Bahia.

In this study, the infected *N. lasiurus* was collected from the soil stratum of the forest region of area 1, corroborating studies that describe its habitat use as being terrestrial [14,34]. Although area 1 is located far (11 km) from the urban center (Figure 1), it is an anthropized area, with evident deforestation for timber exploitation and a farm located in the vicinity, the headquarters of which are inhabited by humans with domestic dogs, horses, and cattle. Therefore, the zoonotic cycle of leishmaniasis may be established due to the alteration of these forest environments, bearing in mind that phlebotomines fly in search of new hosts and small wild mammals and can adapt to domestic and peridomestic environments [30,35,36]. Furthermore, dogs are considered the main domestic reservoirs of the parasite causing visceral leishmaniasis [37].

The evidence of environmental alterations found in area 1 enables direct contact between human beings, domestic animals, and wild animals with vectors transmitting protozoa of the genus *Leishmania*. The broad living area of *N. lasiurus*, which varies from 200 to 2500 m^2^, together with alterations in its habitat, reveals this species of rodent can be present in domestic and wild environments, thereby acting as a link between the cycles of the disease in these two environments. In addition, this animal species is a potential source of food for peridomestic phlebotomines such as *Lutzomyia intermedia* and *Lu. whitmani* [30,34].

The present study demonstrated the presence of *Leishmania* spp. in the internal organs of *N. lasiurus*. Thus, according to Roque & Jansen [12], this rodent species may be contributing to the maintenance of the parasite in the region in which it was found, acting as a maintenance host. However, future studies are needed to clarify whether these animals can act as reservoirs of the agent.

*Leishmania* spp. detection in marsupials has been described in various studies [7,12,17,32,38,39,40]. However, to date, there have been no reports in the literature of natural *Leishmania* spp. infection in individuals of the species *G. microtarsus.* Therefore, this is the first description in Brazil, with 50% (12/24) positivity for *Leishmania* spp. (Table 3), 25% (3/12) of which is due to infection with *L. infantum* (Table 4). These individuals were collected in the soil and arboreal strata of the forest region. This corroborates the study that describes *G. microtarsus* as an essentially arboreal marsupial with vertical habitat displacement that mainly occupies the upper layers of the forest, with possible occurrence in the understory or even on the ground [41]. These characteristics enable the affirmation that the vector is circulating in one of the two forest strata or both, and that these animals were, necessarily, infected in the interior of the forest since they do not move horizontally.

The marsupials infected with *L. infantum* were collected in areas 1, 6, and 7, located in the municipalities of Itambé and Itapetinga (Figure 1). Although these municipalities are not endemic for VL, an autochthonous case of human disease was recorded in Itapetinga in 2018 [42]. It should be noted that all the animals infected with *Leishmania* spp. and *L. infantum* were collected in the same year. These data confirm the presence of the vector and the parasite in the studied region. In addition, the evidence suggests the transmission cycles are independent for each area since the minimum distance between the sampling areas is 8 km, making it impossible for the phlebotomine to fly from one area to another, as its flight extension is a maximum of one kilometer [43].

*Lutzomyia longipalpis* is the main vector of *L. infantum* and is found distributed across almost the entire state of Bahia. Moreover, it was the most prevalent phlebotomine species (42.78%) recorded between 2009 and 2012 [44] (Rodgers et al., 2019), with occurrences in the municipality of Itapetinga [45]. Studies indicate that the proliferation of phlebotomines is greater in the rainy season [30]. In this regard, ecological studies on phlebotomines in Bahia have shown that the population density of these insects, especially *L. longipalpis*, increases after the end of the rainy season [46]. The 13 animals found to be infected in this study were collected during the dry season, the time at which the proliferation of insects is lower. However, this does not mean that infection occurred in this season, since the course of the disease can be prolonged.

Although dogs are considered the main reservoir of *L. infantum* in Brazil, the role of the marsupial *Didelphis* spp. as a wild reservoir has been suggested by various authors, thus emphasizing that the synanthropy of these animals aids the connection between wild and peridomestic environments [12,38,39,47,48]. The fact that *Leishmania* spp. DNA has been found in a significant number of individuals of the species *G. microtarsus* is a strong indication that these animals may act as sources of infection for the vectors. However, further studies would be necessary using xenodiagnosis with individuals of this species and the competent vectors of the region to prove its infectiousness for said vectors [7].

Finally, these results suggest that the human beings inhabiting houses situated close to the forest fragments in areas 1, 6, and 7 are susceptible to infection with VL, since *L. longipalpis* females are able to cover distances from 250 m to 1 Km [43] and have been found in intradomiciliary and peridomiciliary environments [9]. These areas are anthropized, with evidence of deforestation, with houses and the constant presence of humans, dogs, and other domestic animals in their interiors and surroundings. According to Quinnell & Courteney [4], the wild and peridomiciliary transmission cycles, as well as their wild and domestic hosts, are related, act together, and intensify the transmission of the agent to human beings [4].

The possibility of at least two species of wild mammals of different genera having participated in the *Leishmania* spp. transmission cycle in the studied region demonstrates the complexity of the enzootic cycles of this parasite, thus creating an enigma with several peculiarities yet to be discovered [12]. Finally, the anthropization of the areas sampled in this study may modify the pathogen transmission dynamic and lead to the appearance of zoonoses, such as leishmaniasis [49,50].

## 5. Conclusions

This study presents noteworthy findings regarding the detection of *Leishmania* spp. infection in *Necromys lasiurus*, representing the first documented case of natural infection in this rodent species in Bahia state. Furthermore, it reveals the natural infection of *Gracilinanus microtarsus* with *Leishmania* spp. and *L. infantum*, signifying the first recorded occurrence of leishmaniasis in this marsupial species in Brazil. These findings highlight the circulation of the parasite among small wild mammals in fragments of the Atlantic Forest, underscoring the potential risk of *Leishmania* spp. transmission to humans and domestic animals with direct exposure to these forest remnants in the region.

Despite these notable findings, the primary concern lies in the alarming deforestation rate in the region. Although the forest remnants are highly fragmented, they still support native fauna that are part of the parasite’s wildlife cycle. However, if these Lilliputian fragments continue to be deforested, the interaction between wildlife, domestic animals, and humans will intensify, thereby increasing the risk of transmitting this zoonotic disease.

## Figures and Tables

**Table 1:** Species of small mammals, according to their taxonomic classifications, collected from the areas of Atlantic Forest remnants and pastures in the municipalities of Itapetinga and Itambé, in the Southwest region of Bahia, Brazil.

Taxon	Area	N	Positives
**Order Didelphimorphia**			
Family Didelphidae			
*Didelphis albiventris* (Lund, 1840)	All	25	0
*Gracilinanus microtarsus* (Wagner, 1842)	1; 4; 5; 6; 7	24	12
*Monodelphis domestica* (Wagner, 1842)	7	1	0
*Marmosops incanus* (Lund, 1840)	1; 3; 4; 6; 7	58	0
**Order Rodentia**			
Family Cricetidae			
*Calomys expulsus* (Lund, 1841)	All	87	0
*Calomys* cf. *tener* (Winge, 1887)	2; 4	2	0
*Cerradomys vivoi* (Percequillo, Hingst-Zaher & Bonvicino, 2008)	All	108	0
*Necromys lasiurus* (Lund, 1841)	All	74	1
*Oligoryzomys* cf. *flavescens* (Waterhouse, 1837)	3; 4; 6	3	0
*Oligoryzomys nigripes* (Olfers, 1818)	1; 3; 4; 5; 6; 7	48	0
*Rhipidomys mastacalis* (Lund, 1840)	1; 2; 4; 6	15	0
Total		445	13

N: total number of collected individuals.

**Table 2:** Number of infected animals and infection prevalence in the sampling areas, and their respective distances from the urban centers and geographical area.

Area	Negative	Positive	Total	Prevalence%; 95% CI	Extension (ha)	Distance from the Urban Center (km)
1	66	3	69	4.34; 1.43–13.1	283	11
2	61	0	61	0.00; 0–0	992	10
3	52	0	52	0.00; 0–0	91	9
4	70	0	70	0.00; 0–0	99	5.5
5	55	0	55	0.00; 0–0	98	2.5
6	54	6	60	10; 4.68–21.3	85	11
7	74	4	78	5.12; 1.97–13.3	542	15

CI: confidence interval.

**Table 3:** qPCR results for *Leishmania* spp. in the rodents and marsupials collected in the different areas.

Host Species	Positives/N	Prevalence%; 95% CI	Areas
Rodentia	1/337	0.3; 0–2.1	
*Necromys lasiurus*	1/74	1.3; 0.1–9.4	1
Didelphimorphia	12/108	11.1; 6.5–18.9	
*Gracilinanus microtarsus*	12/24	50.0; 33.5–74.5	1; 6; 7

CI: confidence interval.

**Table 4:** Animals positive for *L. infantum*, their respective areas, their identification number in GenBank, and similarity percentages.

Identification Number	Species	Areas	Genbank	Similarity Percentage
27	*G. microtarsus*	1	XR_001203206	100%
42	*G. microtarsus*	6	KY707970	100%
345	*G. microtarsus*	7	XR_001203206	100%

## Data Availability

The raw data supporting the conclusions of this article will be made available by the authors upon request.
